# Lemmel’s Syndrome Secondary to Common Bile Duct Compression by an Inflamed Duodenal Diverticulum

**DOI:** 10.7759/cureus.16959

**Published:** 2021-08-06

**Authors:** Ashley R Gao, Abhishek Matta, Rishi Seth, Dinesh Bande

**Affiliations:** 1 Internal Medicine, University of North Dakota School of Medicine and Health Sciences, Fargo, USA; 2 Hospital Medicine, Sanford Health, Fargo, USA; 3 Internal Medicine, Sanford Health, Fargo, USA

**Keywords:** lemmel’s syndrome, duodenal diverticulitis, obstructive jaundice, extraluminal duodenal diverticulum, ercp, hyperbilirubinemia, eus, biliary obstruction, cholestasis

## Abstract

A 50-year-old female presented with acute epigastric abdominal pain, diarrhea, nausea, and vomiting for two days. Laboratory data showed hyperbilirubinemia and leukocytosis. Abdominal imagining was concerning for a pancreatic head/uncinate process lesion concerning a pancreatic neoplasm. Subsequent esophagogastroduodenoscopy with endoscopic ultrasound and endoscopic retrograde cholangiopancreatography found the major papilla adjacent to an inflamed and infected duodenal diverticulum, which was extrinsically compressing the distal common bile duct, causing biliary obstruction and common bile duct dilation. These findings are consistent with a diagnosis of Lemmel’s syndrome. A biliary sphincterotomy was performed to relieve the obstruction and one temporary plastic biliary stent was placed into the common bile duct. The duodenal diverticulitis was treated with antibiotics for 10 days and the patient made a good recovery.

## Introduction

Lemmel’s syndrome is characterized by extrinsic compression of the distal common bile duct by a periampullary diverticulum, causing obstructive jaundice in the absence of choledocholithiasis or tumors of the pancreaticobiliary system [1]. The duodenal diverticulum can also cause sphincter of Oddi dysfunction, leading to pancreatitis and cholangitis. Currently, there are very few case reports of this condition, which was first named in 1934 after Dr. Gerhard Lemmel [1].

Although noninvasive testing, such as CT scans, can support diagnosis, endoscopic retrograde cholangiopancreatography (ERCP) or endoscopic ultrasound (EUS) remain the preferred diagnostic tool [1,2]. Given the rarity, it is important for clinicians to utilize history and physical exams, aided by ERCP, in order to promptly recognize and treat Lemmel’s syndrome [1]. We describe a case of Lemmel's syndrome to emphasize the importance of imaging studies and endoscopy in the diagnosis and management.

## Case presentation

A previously well 50-year-old female, with a past medical history of moderate persistent asthma and depression, presented with acute epigastric abdominal pain associated with diarrhea, nausea, and vomiting. She reported having an episode of explosive diarrhea one hour prior to the onset of the abdominal pain. The pain was described as a cramping sensation, without radiation. She was febrile with a temperature of 100.6^o^ F, blood pressure of 151/79 mmHg, and heart rate was 72 beats per minute. On physical examination, the abdomen was soft and flat, with mild tenderness in the epigastric and right upper quadrant. Murphy’s sign was negative. Although she had mild hyperbilirubinemia, she did not display scleral icterus and her skin was not jaundiced.

At the time of admission, the patient was found to have leukocytosis, elevated liver enzymes, C-reactive protein, and lactic acid (Table 1).

**Table 1:** Laboratory data on admission

Laboratory Test	Value	Reference Range
White Blood Cells (WBC)	15.3	4.0-11.0 K/µL
Total Bilirubin	2.1	0.2-1.2 mg/dL
Direct Bilirubin	0.5	0-0.4 mg/dL
Alanine Aminotransferase (ALT)	116	0-55 U/L
Aspartate Aminotransferase (AST)	164	0-35 U/L
Alkaline Phosphatase	69	30-150 U/L
C-reactive Protein (CRP)	31.4	0.0-8.0 mg/L
Lactic Acid	2.9	0.5-2.2 mmol/L

Alkaline phosphatase and lipase remained within normal limits. The viral hepatitis panel was nonreactive, and urinalysis was unremarkable.

**Figure 1:**
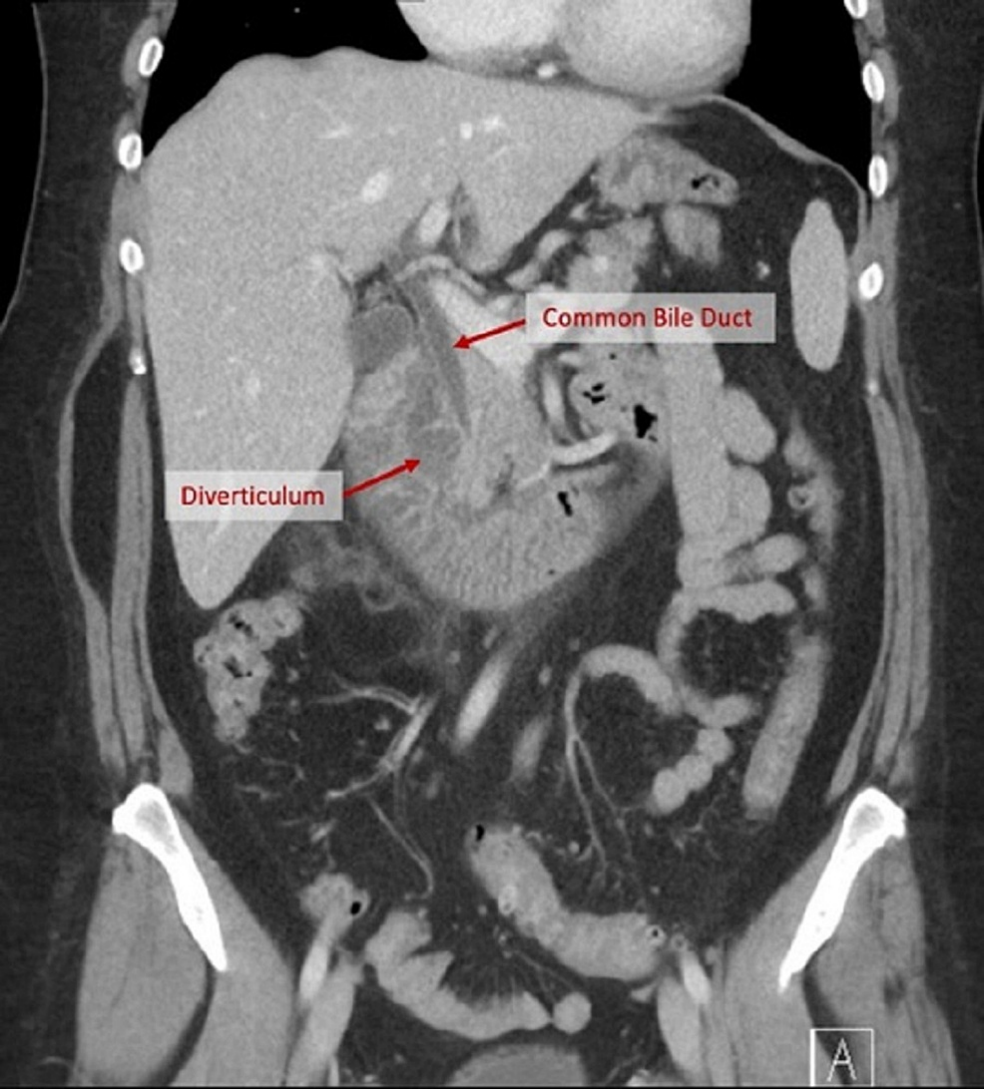
CT abdomen and pelvis showing the common bile duct (CBD) and duodenal diverticulum

CT abdomen and pelvis showed peripancreatic and periduodenal fat stranding involving the head/uncinate process of the pancreas and descending duodenum. A hypoattenuating lesion in the posterior aspect of the pancreatic head/uncinate process measuring 3.0 x 1.8 x 2.8 cm was concerning for a pancreatic lesion (Figure 1).

**Figure 2:**
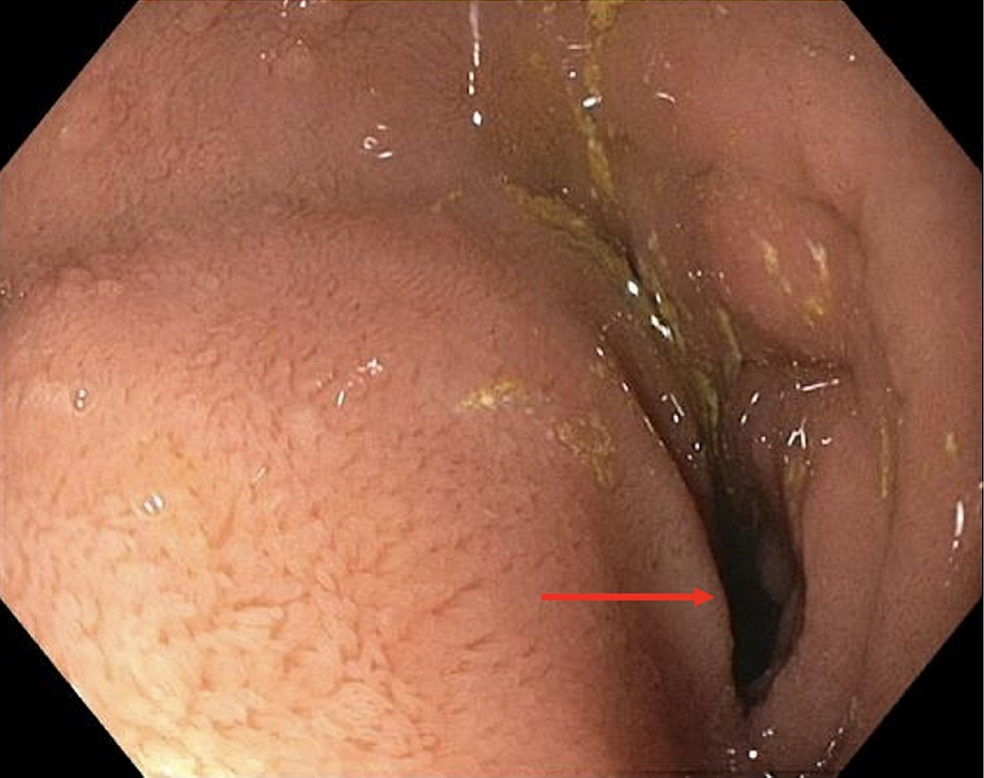
Endoscopy image of the duodenal diverticulum (arrow)

**Figure 3:**
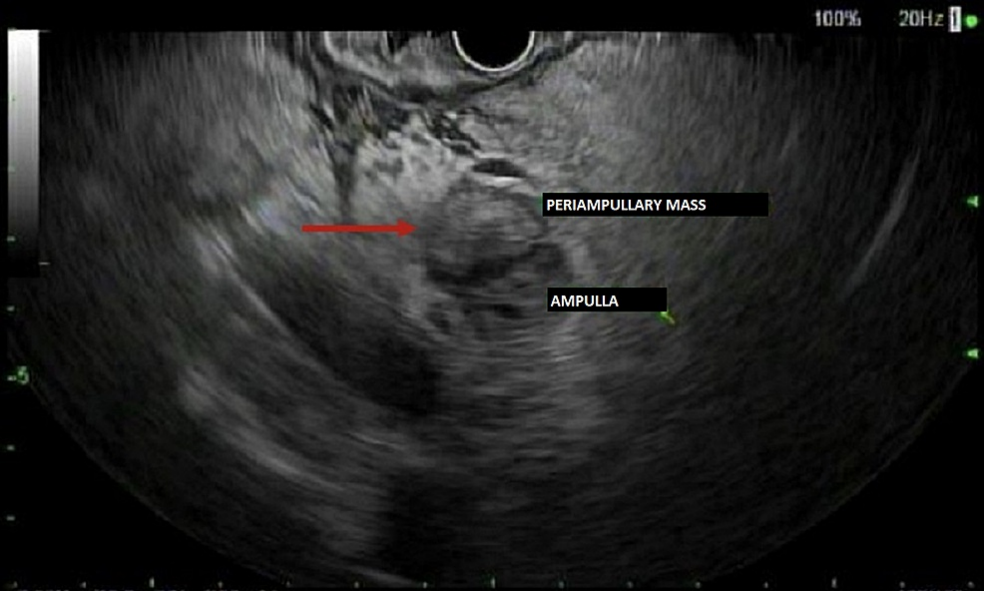
EUS showing periampullary diverticulum EUS - endoscopic ultrasound

She subsequently underwent esophagogastroduodenoscopy (EGD) with EUS and ERCP. The major papilla was found to be adjacent to a diverticulum (Figure 2), which was extrinsically compressing the distal common bile duct, consistent with Lemmel’s syndrome. EUS confirmed the periampullary diverticulum (Figure 3).

 

**Figure 4:**
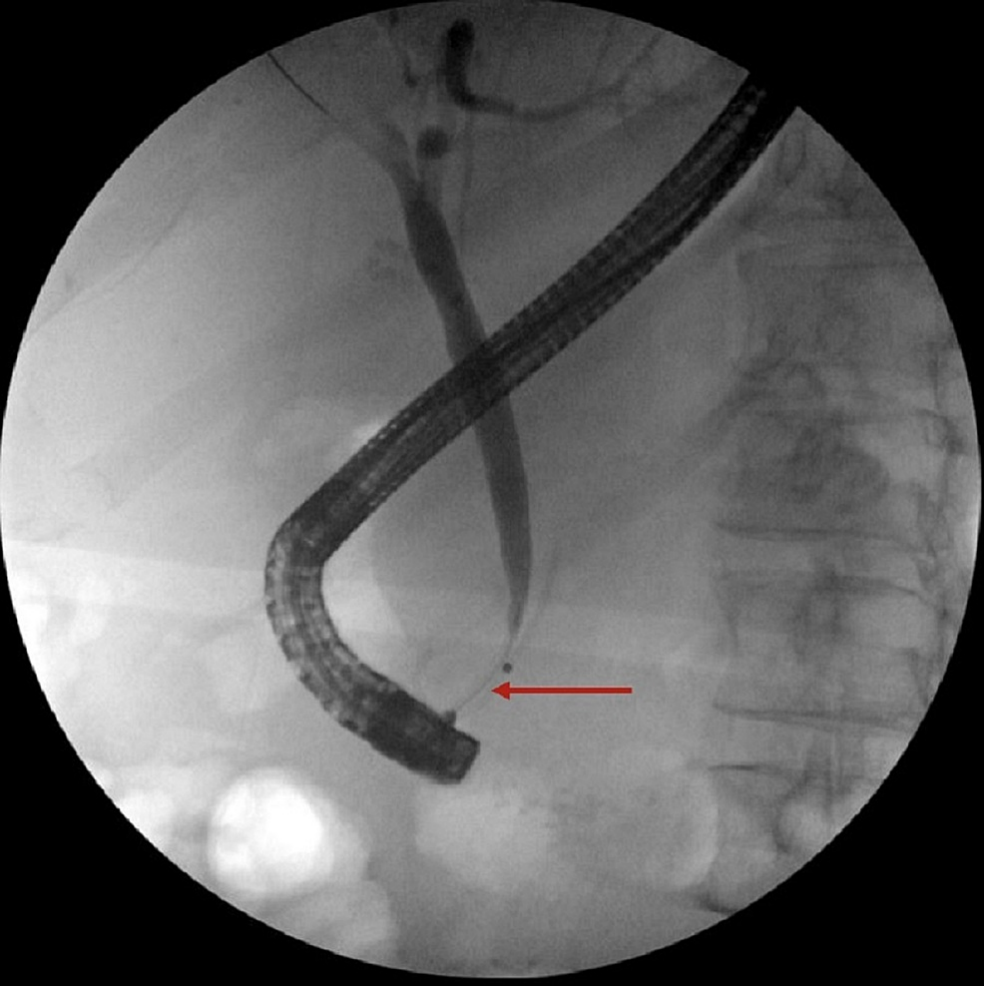
ERCP showing distal CBD compression ERCP - endoscopic retrograde cholangiopancreatography; CBD - common bile duct

The diverticulum was cleaned out, producing a copious amount of purulent fluid suggestive of acute diverticulitis. The main bile duct was dilated up to 8 mm. The biliary pancreatic junction contained single localized stenosis less than 5 mm in length, smooth appearing, confirming Lemmel's syndrome (Figure 4).

A biliary sphincterotomy was performed to relieve the obstruction and one temporary plastic biliary stent was placed into the common bile duct, to be removed in three months. Fortunately, no concerning pancreatic head mass was noted on EUS and there was no evidence of significant pathology in the visualized portion of the liver. No cholelithiasis or choledocholithiasis was noted. Her blood cultures were also monitored and negative after five days of growth.

The patient was started on intravenous ceftriaxone and metronidazole for duodenal diverticulitis before switching to oral ciprofloxacin and metronidazole at discharge, for a total antibiotic duration of 10 days. At her follow-up appointment nine days after discharge, the patient reported mild fatigue but was back at work. Vital signs were within normal limits and other than mild, diffuse abdominal tenderness, her physical exam was unremarkable. The plan is to remove her biliary stent three months later.

## Discussion

Lemmel’s syndrome is the extrinsic compression of the distal common bile duct by a periampullary diverticulum, causing obstructive jaundice in the absence of choledocholithiasis or tumors of the pancreaticobiliary system [1,2]. First described in 1934 by Dr. Gerhard Lemmel, Lemmel’s syndrome mimics other pancreaticobiliary diseases, making diagnosis very challenging [1]. Currently, there are very few case reports of this syndrome.

Duodenal diverticula are more commonly found in adults age 70 or older and have a prevalence of 22% in the general population; however, the detection rate varies depending on imaging modalities [1,3,4]. About 75% of duodenal diverticula are found in the descending duodenum, adjacent to the ampulla of vater [4]. Periampullary diverticula can be extramural or intramural, and most are extraluminal giving it ample opportunity to compress the common bile duct [2,3].

The majority (95%) of duodenal diverticulum are asymptomatic and are usually found incidentally during endoscopic procedures. Up to 5% can become symptomatic and/or lead to complications such as hemorrhage, fistula, and perforation. Extrinsic compression of the common bile duct can give rise to complications such as obstructive jaundice, acute pancreatitis, and bacterial overgrowth leading to cholangitis [2,3].

Noninvasive imaging, such as CT scans, may show periampullary diverticula as a thin-walled cavitary lesion off the medial aspect of the descending duodenum, thus resembling pancreatic pseudocysts, abscesses, or neoplasms [2,5,6]. Side-viewing endoscope during ERCP is currently the gold-standard test for diagnosis [3,4]. 

Lemmel’s syndrome can be treated by endoscopic sphincterotomy with biliary stent placement or diverticulectomy [1,5]. More research may need to be done to assess recurrence rates with sphincterotomy versus diverticulectomy. While Lemmel’s syndrome does not necessarily increase the risk of pancreaticobiliary malignancy, missed diagnosis of Lemmel’s syndrome may lead to recurrent pancreatitis and cholangitis, which poses a risk of malignancy and increased morbidity and mortality [5]. Given the risk of complications and recurrence, it is important for clinicians to quickly diagnose and manage these patients.

## Conclusions

Lemmel’s syndrome is the extrinsic compression of the distal common bile duct by a periampullary diverticulum, causing obstructive jaundice in the absence of choledocholithiasis or tumors of the pancreaticobiliary system. Biliary sphincterotomy with stenting during ERCP provides conservative management of obstructive jaundice. Recurrent cholangitis and/or pancreatitis may warrant surgical intervention for the periampullary diverticulum. Given the rarity of Lemmel’s syndrome, it is important for clinicians to utilize clinical findings, aided by ERCP, in order to promptly recognize and treat Lemmel’s syndrome.
